# “Interactive Technology Assessment” and Beyond: the Field Trial of Genetically Modified Grapevines at INRA-Colmar

**DOI:** 10.1371/journal.pbio.1000551

**Published:** 2010-11-30

**Authors:** The Local Monitoring Committee, Olivier Lemaire, Anne Moneyron, Jean E. Masson

**Affiliations:** INRA/UDS, UMR1131 Santé de la Vigne et Qualité du Vin, F-68021 Colmar, France; London School of Economics and Political Science

There is increasing interest worldwide in how decision-making processes concerning controversial technological innovations can be improved by better integration of input from society at large. Aiming to gain knowledge on grapevine defence mechanisms against grapevine fanleaf virus (GFLV), a severe disease, scientists wanted to carry out a field trial of genetically modified grapevine rootstocks in Alsace. Three millennia of winemakers' tradition and innovation suddenly collided in this highly reputed cradle of French viticulture. The French Institut National de la Recherche Agronomique (INRA) used the Interactive Technology Assessment (ITA, Box 1) method in an initial consultation phase to integrate public input and then went beyond ITA to carry out the field trial. The implementation phase was made possible by the involvement of a Local Monitoring Committee (LMC, Box 2) with broad stakeholder representation. In the course of 7-years' work, the LMC built a research-action program which allowed redesigning of the initial GM grapevine research assay, as well as developing innovative trials on environmental impact and organic viticulture.

**Box 1: Interactive Technology Assessment**

Interactive Technology Assessment [4] consists in ensuring the interaction of three “worlds”: research, the profession (industry), and civilian society. The idea is to set up a small group (in practice, 12 to 14 people) to provide a forum for discussion. The ITA's leader chooses membership of the group relying on criteria that reflect different visions of the world and different professional backgrounds. The group focuses on the subject, develops the questions it wishes to raise, explores the different dimensions of the problem, and compiles a report. At the end of its work, the report and/or opinion serve as a support for decision makers [5]–[7].

**Box 2: The Local Monitoring Committee**

The Local Monitoring Committee comprises members from the Alsace winegrowing profession, the Association of Alsace Winegrowers, the Institut National des Appellations d'Origine, the Alsace Consumers Association, the Agricultural and Viticultural Training School, an agricultural union (la Confédération Paysanne), a nature preservation society (Alsace Nature), an independent winegrower, a neighbour of the trial site, a representative from the Plant Protection Services (DRAAF), a representative from the Regional Directorate for the Environment (DIREN), an elected representative from the Regional Council, an elected representative from the Town Council, the researcher managing the research programme and the President of the INRA Centre in Colmar, who acts as moderator. In contrast to the usual mode of composition of committees, membership in the LMC was spontaneous. Members of the CLS attended each meeting without preparing any proposals or objections in advance, and without intending to force any viewpoint. Each meeting was the subject of an exhaustive report on its discussions. During the six years of effort and reflection, the LMC members referred to their commitment (which remained unchanged during this period) as a “motivation,” experiencing the issues to be addressed as singular and complex: “these are subjects that are anything but simple”; “this is not a subject like any other, that is why we are here!”; “Today, we could be at several other meetings starting at the same time, but we are here!”; “The challenge, and the motivation, for us is that everything starts here.”

**ITA versus LMC**

The initial ITA was a short-term consultation which let the committee members express themselves and properly describe obstacles and possible issues to INRA research projects on grapevine. Concerning the GMO root-stocks trial, the ITA report suggested that it should be followed by a local monitoring committee ([6],[7]; http://www.inra.fr/la_science_et_vous/ogm). The LMC, in the course of 7-years' work, defined its own route, notably while re-designing the initial research project and diversifying research objectives, which was not within the intents of the ITA. The LMC headed a research–action program, which can be considered as a type of PTA (Participative Technology Assessment).

Over the past decades, society in Europe has experienced repeated crises highlighting cross-relationships between health, politics, science, agriculture, the environment, and society. The immediate effect of these unprecedented crises has been the growth of doubt, and “the collapse of certainties linked to the idea of guaranteed progress, and collapse of the idea that science and technology can only be beneficial has introduced the worm of uncertainty everywhere” [1]. This change in public attitude in Europe is perfectly illustrated by the acceptability of genetically modified (GM) plants. During the late 80s and early 90s, Europe was a hotbed of research and development concerning GM plants, and numerous field trials were performed without significant public opposition. More recently, attitudes in many European countries have become distinctly anti-GM, as shown by the repeated destruction of field trials of GM crops. Years after the directive 2001/18/EC of European Parliament, the political posture of European countries has still not settled [2].

Nonetheless, there are certain cases where genetic engineering is one of the few means available to solve a major agronomic problem or to study fundamental mechanisms of plant biology, as a first step. For instance, Grapevine fanleaf virus (GFLV), which is transmitted by soil nematodes, is the cause of an often lethal disease of grapevines with worldwide distribution. Until recently, controlling the disease typically involved removing infected individuals and treating the soil in and around the focus of infection with nematicide fumigants, but this is no longer possible, since these nematicides were banned due to their unacceptable environmental impact. For this reason, researchers at the French Institut National de la Recherche Agronomique (INRA) and several other labs developed potentially GFLV-resistant grapevine rootstocks, on which non-GM scions of the traditional grape cultivars could be grafted [3]. Since the virus is soil-transmitted, the resistance of the rootstocks should be sufficient to protect the scions, thus making it possible to make non-GM wine from the grapes borne by the scions.

Following the authorization of the French Ministry of Agriculture, and based on opinion of the Biomolecular Engineering Commission (B/FR/94-11-04), a first GM rootstock trial was set up in 1996 on a grapevine plot in the Champagne region that was affected by GFLV. Analyses performed on the plot suggested that the GM rootstocks could delay the onset of GFLV infection by at least three years [3] (Figure 1). The researchers wanted to confirm these preliminary results, to determine the mechanism of this resistance to the disease, and also assess its sustainability and environmental impact. However, in a period of increasing hostility towards GM organisms, particularly in France, an article entitled “Des bulles OGM dans le champagne” (1999) (“GM bubbles in Champagne”) was published by a prominent weekly satirical paper, the Canard Enchaîné. This led INRA's partners to abandon the project because they did not want to face the issues of public acceptability of wine made from grapes borne by partly GM grapevines. This put an end to the first field trial, and raised the question of whether it would be possible to continue elsewhere, and if so under what conditions.

**Figure 1:**
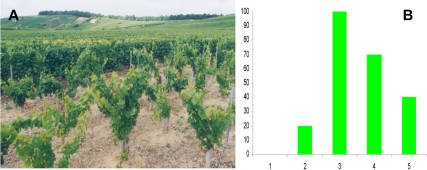
The first GM grapevine rootstock trial in a vineyard, 1996–1999. (A) Chardonnay plants grafted onto standard or GM rootstock planted in a Champagne vineyard affected by grapevine fanleaf virus. (B) The proportion of GFLV-free Chardonnay plants after 4 years of the trial. Plants grafted onto standard rootstock [1] and GM rootstock [2]–[5].

## The Colmar GM Grapevine Rootstock Field Trial

In spite of public opposition, particularly regarding what is certainly the most socially emblematic crop in France, INRA decided to persist in this project, but under quite different circumstances. However, this move was consistent with the issues addressed by this public-sector mission-oriented research organisation and with the research projects that it carries out. It was in this context that INRA's central management stipulated that “field trials, carried out with parsimony and precautions, are sometimes essential to test and verify the behaviour of GM organisms in a complex environment that cannot be reproduced in a glasshouse” (www.inra.fr).

To deal with these challenges, a discussion and working group was set up in 2001. Its operation was based on the Interactive Technology Assessment (ITA) method [4], and its work focused on issues of innovation in grapevines and wine [5]–[7]. Following this first stage of consultation, in the spring of 2003, a Local Monitoring Committee (LMC) was set up for the GM grapevine rootstock trial at the INRA Centre in Colmar. Its mission was defined thus by INRA's central management: “the trial protocol shall be determined by scientists and then discussed by a Local Monitoring Committee and rendered public” [6].

Because of the increasing complexity of the issues raised and the Committee's acceptance to deal with them, the LMC in fact came to act in a very different way. It thus became a group that developed proposals and forecasts and built up a research–action programme. During the first working meeting of the LMC in 2003, one of its members, a winegrower, expressed his concerns about the possible environmental impact of this trial, wondering, “Is it possible that by boring into the roots of the GM rootstock, the nematode could absorb a gene and then disperse it into the environment?” The researcher who was at that time responsible for the trial responded: “What you suggest is not possible, this question is not one to be addressed, scientifically it makes no sense.” The first real question had been raised and would give rise to a new definition of the committee's working practices.

As a consequence of this first question and response, the President of the Colmar Centre, acting as the moderator of the meetings, triggered a radical change of approach. He declared that in the context of this committee, and regardless of who was speaking “we shall listen to everything, and try together to answer the questions raised by jointly constructing research that might provide scientific answers to these questions.” From that moment, the paradigm of the relationship between learning and scientific knowledge shifted. The issues debated concerned the most important and controversial aspects of this trial: its environmental impact, the image of grapevines and wine, and the regional identity of Alsace. Because of their complexity, these issues could have become insurmountable obstacles. These did not initially result from a hostile fear of change [8] but were based on knowledge gained from long-term experience with successive crises in the winegrowing community. It was important to take into account the intuitive fears expressed and this unspoken knowledge because it opened the way to the expression of emotions, a dimension that is now known to form an integral part of knowledge [9]. By basing its work on the “three fundamental aspects of a cross-disciplinary approach: rigour, openness, tolerance” [10], the LMC gradually started to develop its research–action method.

Starting at the first meeting, the image of grapevines and wine, in their regional and traditional context, came up against the image of INRA research, which signifies modernity and progress. Nevertheless, because of the public's desire for strong public-sector research, these two antagonistic images remained equally important and valid in the eyes of all. Throughout the lengthy time frame of its working process, the LMC wished to produce knowledge through the joint development of pertinent research issues, as expressed by Edgar Morin: “knowledge is a multidimensional phenomenon, insofar as in an inseparable manner it is physical, biological, cerebral, mental, psychological, cultural and social” [11].

The fact that the GM rootstock trial was based at the INRA site in Colmar might appear to have been an obvious decision because of the logistical convenience for the research institution, whereas in fact this was not the case. When it came to choosing a site for the trial, it was necessary to face up to the wine-growing profession's refusal to allow the trial to take place on land within the officially recognized perimeter of Alsace vineyards and, more generally, its rejection of GM organisms. It also revealed a collective denial by the profession that GFLV has a major impact. News of rejection of the trial spread throughout the Alsace region, to the point that the only possible site was at the INRA Centre. However, since the Centre is not affected by GFLV, the trial had to be performed with soil brought in from a GFLV-affected vineyard. In this context, finding soil from a GFLV-affected vineyard meant that a winegrower had to acknowledge the existence of the disease in his grapevines as well as his inability to control it using his own knowledge. Furthermore, French winegrowing regulations governing *appellation contrôlée* wines required that the soil removed for the trial should be replaced by soil from the same appellation but free of disease. It was thus necessary to find two winegrowers from the same appellation and the same small area who would be prepared to commit themselves to the project. There was in effect a broad ideological confrontation between researchers and the winegrowing community that needed to be acknowledged and overcome. The scientists were convinced of the importance of their research, while the winegrowers—because of the choice of GM organisms as a means of control—stated they had other priorities in terms of grapevine diseases (those affecting wood, etc.). So the sites and the affected soil were difficult issues in a collective and individual front that was both objective and subjective and had to be redrawn.

**Figure 2:**
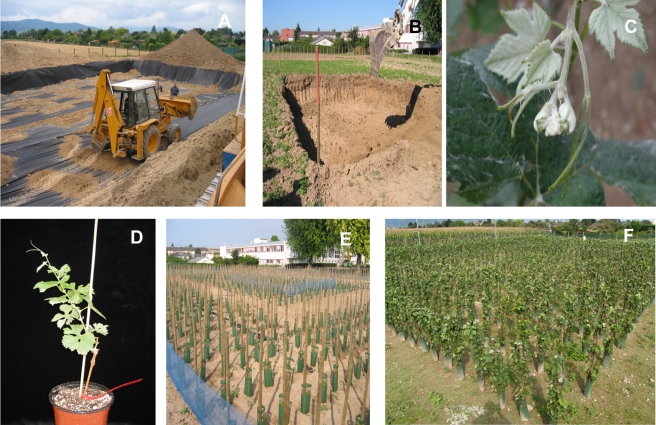
Construction of the experimental site and initiation of the trial in Colmar. (A) Laying of the microporous geotextile membrane. (B) After filling with soil from the site, preparation of the area that will receive soil from the GFLV-affected vineyard. (C) Leaves of the Pinot Meunier variety. (D) Pinot Meunier plant (leaves and stem) grafted on a GM rootstock (trunk). (E–F) The trial after planting in September 2005 and in 2008, respectively.

Only the grapevine scion can produce flowers, and none of the scions was of GM origin during this trial. Nevertheless, for uninformed visitors, the time given to images would be greater than that allowed for explanations. Thus, seeing flower heads would have inevitably associated this trial with questions linked to the dissemination of genes. Although grapevine is an easily recognisable plant, the general public tends to distinguish grapevine varieties in terms of the wines they produce and only with difficulty in terms of the plants. The LMC thus chose to graft onto the rootstocks a variety, Pinot Meunier, that does not exist in Alsace and has a distinctive appearance because the leaves are so downy that they appear to be white in the sun (Figure 2).

The LMC also chose to eliminate all flower heads from the 1,588 plants involved in the trial, although only 70 of them were grafted onto GM rootstocks. For the INRA researchers, these choices further complicated their building on the results of the initial trial performed in Champagne, but they were decisive for the LMC, as they acknowledged the importance of the image of vines and wine.

## The LMC as Initiator of Further Research

**Figure 3:**
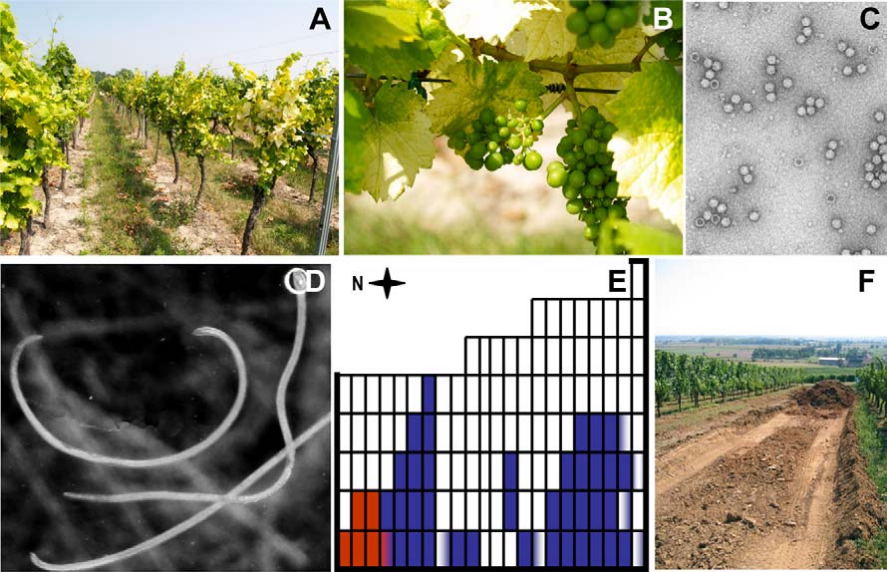
The vineyard plot that donated GFLV-affected soil. (A) Symptoms of GFLV on whole plants, (B) on leaves and bunches of grapes. (C) GFLV particles purified from grapevines, and (D) nematodes in the soil. (E) Measurement of the infective potential of soil of the donor vineyard: presence of nematodes (*Xiphinema index* in blue, *Xiphinema diversicaudatum* in red) in the affected plot. (F) Sampling soil containing nematodes and transport to the trial site.

**Figure 4:**
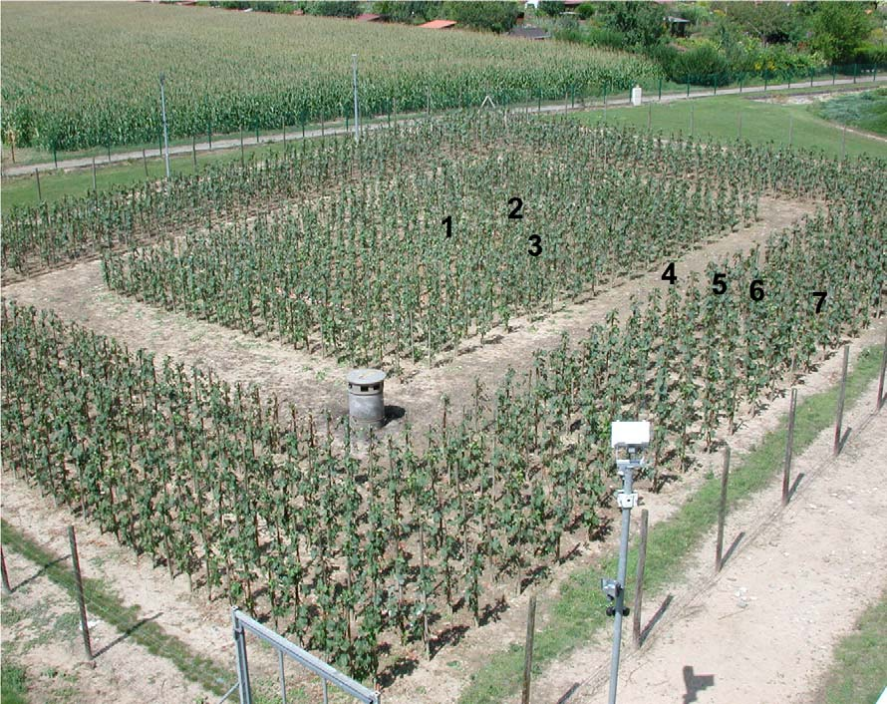
The different cultivation zones of the Colmar GM rootstock trial, including a biomonitoring system. (1) Soil in this zone (40m^3^) came from a vineyard affected by GFLV. The nematodes (20/kg of soil) bore GFLV, and the intention was that they would transmit it to the grapevines by feeding on their roots. Analyses performed on Pinot Meunier grafts made it possible to determine whether GM rootstocks (50 plants) could protect the grapevines compared with standard rootstocks. Zones 2 to 7 contained soil from the INRA site that was free of GFLV virus. (2) Growth of the grapevines grafted on GM rootstock (20) could be compared with that of standard plants in the absence of disease. (3) The detection of standard, affected plants enabled monitoring of the horizontal movement of nematodes from zone 1 over time. (4) If the nematodes colonised all of zone 3, they would not be able to cross zone 4 because there were no roots on which to feed. (5) Thus if plants in this zone remained free of disease, they would testify to the halt in nematode movement. (6) A double, microporous membrane would limit the movement of nematodes (which follow root development) outside the experimental site. (7) Standard grapevines in this zone were there to testify to the absence of nematode migration.

In response to the question raised by the LMC before the trial was put in place regarding the ability of nematodes to transmit genes, a microporous geotextile membrane was buried beneath the plot in order to contain the nematodes and isolate the trial (Figure 2). The roles of the different parts of the trial were discussed by the LMC (Figures 2–4), and a molecular analytical method was developed to characterise the genome sequence of the viruses present in a single nematode [12]. This step was the first demonstration that consideration of the expectations and questioning of society via the LMC can lead to the development of innovative science.

From the start of this project, INRA committed to ensuring that “neither flowers, nor grapes will be produced” on the trial plot, in order to dissociate grapevines and wine from a study dedicated to research in which only the rootstock was of GM origin. However, in 2004, and again in 2006, the LMC returned to this issue with a highly pertinent question of biology: “are there any exchanges of genes between the two parts of the grapevines, between the rootstock and scion?” Although this question focused on GM organisms, its basis goes back more than a century in the history of winegrowing. The first line of defence against the devastation of European vineyards caused by phylloxera, a type of root aphid that was accidentally introduced from America, was hybrids created by crossing susceptible European and resistant American grape species. These direct-producer hybrids were indeed protected against phylloxera, but the wines produced were of such poor quality that they almost wiped out winegrowing in Europe. It was by grafting the ancestral, high-quality varieties onto hybrid phylloxera-resistant rootstock that the quality of wines was restored. Nevertheless, even after decades of use, doubts persist as to whether the hybrid rootstock could transfer factors across the graft junction and thus alter the quality of the wine. In the context of the present project, the LMC agreed that molecular analyses should be performed on the grapevines in order to evaluate the potential for molecular exchanges between rootstock and scion. And indeed, a scientific demonstration of the pertinence of the question of “transfer at the grafting point” has recently been made in research in tobacco [13].

“Are there any exchanges between soil microflora and the GM rootstock?” was a question raised halfway through the trial and to which INRA had no specific answer. The researchers belonging to the LMC committed themselves to monitoring future scientific publications in this area. For instance, the results of a study carried out elsewhere in France, performed at sites used to cultivate GM maize resistant to corn borer were published in 2008. Although, as in previous studies regarding this general question, no exchanges between GM plants and soil bacteria in the field were observed [14], the LMC decided to jointly develop a specific research programme for the trial site and to submit it for funding to the French Agence Nationale de la Recherche.

With the aim of diversifying research on GFLV, the LMC also organised a three-nation symposium on fallow vineyard land. German, Swiss, and French actors in the winegrowing sector; researchers; and winegrowers (organic or not) shared their knowledge on the control of nematodes as vectors of GFLV. Based on these data, the LMC then developed a research programme on the use of fallow periods to control GFLV. This started in the autumn of 2009, and implements organic winegrowing practices that could rehabilitate soils with limited infective potential to below the harmful threshold (www.inra.fr). After seven years work, the most radical actors of collective denial of GFLV disease—organic winegrowers—spontaneously contacted the LMC to be involved in the fallow project. Their commitment, though never intended from LMC's side, suggested global agreement with LMC's way of working as it brought solid scientific data. Actually, preliminary results suggest that plants they used for decades, and still today, to fight the disease and improve soil structure, actually favoured nematodes or/and virus spread (unpublished data). This data pointed out existing conflicts in the winegrowers' community about the validity of organic viticulture practices versus standard practices. Instead of increasing conflict, this disagreement was structured by the LMC and legitimated back science for validation, even for organic practices.

## Science and Society Seen through the Experience in Colmar

Associating science and society raises questions concerning the possible and desirable ways of consulting citizens. Which actors should be addressed and how? For which purposes, and using which participative methods? Where, in geographical terms? On which issues should citizens be consulted? At which times can the different points of view interact? There are various types of contribution, such as citizen or consensus conferences [15] or citizens' juries [16]. In these two cases, the prerequisite for consultation is training of the jury members involved regarding the question under consideration. Scenario workshops [17] propose a variety of possible scenarios that are advanced upstream of the interaction and are not a subject of debate. Interactive technological assessment was developed in Sweden [4], but in such groups, those leading the discussion do not review the detailed content of the subject under discussion [18]. The research–action method experimented by the LMC proved to be a novel approach, based on the principle of both acknowledging the learning of all parties and also the validity of other modes of reasoning, which “demonstrate the need to obtain other approaches to relationships with the world” [19]. It thus opens the area of scientific questioning to new possibilities.

The members of the LMC joined voluntarily, and the membership has remained remarkably stable over the years. The members' motivation has been to favour progress in research on grapevines while preserving the image of grapevines and wine. There was no effort to come to a general agreement on GMOs; in fact, most of the LMC members were opposed to GMOs and remain so today. Remarkably, this underlying disagreement on GMOs did not prevent the LMC from working fruitfully as a group, since its objective was specifically to contribute to the management of the field trial and the design of the associated research projects.

This research–action resulted from the uncompromised involvement of all actors in the LMC. It was only by advancing step-by-step from a disciplinary issue to a cross-disciplinary problem linking science, society, humanity, and nature, and by allowing time for reflection, that this new and shared research space was able to develop. The controversy that provided the foundations for discussion was transcribed into pertinent research questions that could then be addressed by scientists. The group was able to extricate itself from a weak consensus and open the way to “structured dissension,” [20],[21]. If society and research are in an antagonistic relationship, and if this cannot be changed, it is the translation and not the refusal of these conflicts that can generate further innovation, based on the “ecology of action” [*22*]. Scientific facts and values are not opposable, but the interactions between science and society can become the driver for a creative dynamic.

For greater efficacy, the participants in the LMC also decided to form a smaller working party. However, the LMC also opted for circulation of the minutes of its meetings, issuing progress reports on its collective activities to regional political decision makers. Under the same open approach, it continues to respond to questions from society. Two hundred conferences and debates with different audiences were organised in order to publicise progress in its debates and the joint construction of the GM trial protocol, followed by the fallow land research programme. These communication efforts constitute tangible evidence of the mutual “reliance” [23] between society and research. They have allowed the project to benefit from the issues raised by society, which, in turn, has received answers to its questions and information to help it to rethink its position. Thus, at the end of the process it is possible to see a new path opening up, which is shared by INRA and society, and will lead to further research [24].

The LMC is rooted in a specific territory: the vineyards of Alsace. But reference to the territory as a factor contributing to its success must be broadened to its cultural dimension. The fact that the leader–coordinator was from this territory and was an active researcher in biology enabled him to understand what was implied in conversations or in the everyday language specific to this territory and to winegrowing. Through his commitment, the leader–coordinator was able to cross the borders between the different stakeholders represented in the LMC, and could go beyond requests for transparency (a distrustful attitude), towards the joint construction of a need for greater clarity (an attitude of reciprocal recognition) between science and society.

## Conclusions

When the field trial was partly destroyed by an isolated individual who sawed the scions off the rootstocks in September 2009, the LMC reacted rapidly and unanimously by reaffirming its wish to continue working, and for the field trial to be restarted. There were more than 80 articles on the field trial destruction (out of 282 articles on the field trial for the 2003–2010 period), and strong written support for the LMC and for continuing the project was received from many individuals and groups: deputies both in the majority and opposition, including a Green party senator, government ministers, chambers of agriculture, members of the Regional Council, research scientists, university professors, but also an association for organic agriculture, and numerous citizens from all over France. These reactions demonstrate that the various stakeholder groups were well-informed and that this form of dialogue corresponds to their expectations. After this event, field-trial protections were reinforced to diminish risks of individual actions. More importantly, acceptance of the legitimacy of the LMC's action became an element in debates regarding stakeholder involvement; acknowledging the legitimacy of this scheme of science–society interaction suggests its potential usefulness beyond the sole question of GMOs.

INRA requested a prolongation of the trial (B/FR/09.11.01) from the national competent authority, the *haut conseil aux biotechnologies*, of which both its scientific panel and its economic, ethical, and social panel have given a favourable opinion. The government has just given its authorization (http://www.ogm.gouv, decision 10/001), and non-GM scions have been grafted again on the pre-existing rootstocks, since they were still living.

Beyond the initial phase of ITA, the work of the LMC was critical to the trial's success. However, this did require mobilizing considerable human and financial resources. For other field trials, elsewhere and under other circumstances, it may well be impossible to develop a field trial support system of this complexity. Nonetheless, the experience in Colmar demonstrates that proper integration of ideas from diverse stakeholders is a key for integrating a trial in the local community.

## Coda

In August 2010, while this paper was being edited, the restarted field trial was uprooted by 65 activists, including a single winegrower, in a manner that made it impossible to re-graft. This extremely unfortunate event shows that it is impossible to provide absolute physical protection to field trials, and also that even when a trial is guided by a stakeholder group that includes people opposed to GMOs, there will always remain a small proportion of the public that will refuse to enter into a constructive dialogue. However, we should also consider that violence here may in fact reflect a quest for listening, for legitimacy in some other form. In addition to ITA, development of improved Technology Assessment with acknowledged public involvement may be needed. A kind of Participative Technology Assessment (PTA) as suggested in a recent editorial [25] could be a possible response. In this regard, perhaps another key element for progress is suggested by the LMC's shift from the initial science–society debate format to a long-term research action programme relying on strong stakeholder's commitment. Founded on a combination of learning, imagination, sensitivity, and scientific demands, the collective can advance, though often through heated debates, to rise above a binary confrontational mode of “for or against.” The fact that neither side attempted to compromise their views counterintuitively served to enrich the research approach.

Reactions to the second destruction of the trial were again mostly supportive of INRA and the LMC, and in addition the Ministers of Research and Education and of Agriculture visited the field trial and publicly made the commitment to fully fund a restart of the research program. They also insisted that it is important to further develop competences regarding GMOs in public-sector research and, inspired by the LMC's example, also proposed to broaden science–society debates on the scale of all public-sector French Research.

**Box 3. GM plants in agriculture**

The total acreage of GM plants in agriculture worldwide has increased during the past decade and reached 140 millions hectares last year (http://www.gmo-compass.org/eng/gmo/db/). Most GM plants (maize, soybean, rapeseed, cotton, sugar beet, rice) are engineered for herbicide, pest or disease resistance. A new generation of GM plants for “green biotech” is emerging, with the objective of producing biofuels or biological molecules with high value. In the period 1995–2010, over 60 GM-grapevine field trials were conducted in the USA, Canada, South Africa, Australia, and Chile. In the same period, Germany and Italy ran five trials and France two. In contrast to the worldwide situation, the surface devoted to GM field trials has declined sharply in Europe, except in Spain. Strong public rejection and repeated field trial destruction by activists in France, Spain and Germany severely hampered studies of GM crops.
